# Ocular sarcoidosis, to screen or not to screen?

**DOI:** 10.3389/fmed.2024.1348435

**Published:** 2024-02-19

**Authors:** Sanna Leinonen

**Affiliations:** ^1^Faculty of Medicine and Health Technology, Tampere University, Tampere, Finland; ^2^Tays Eye Centre, Tampere University Hospital, Tampere, Finland

**Keywords:** sarcoidosis, ocular sarcoidosis, uveitis, sarcoidosis-related uveitis, screening

## Abstract

Ocular sarcoidosis most commonly presents with symptoms and is first diagnosed before systemic sarcoidosis in at least half of the patients with sarcoidosis. Prevalence of ocular involvement in sarcoidosis varies between 2–80% depending on the study setting, included ocular diseases, and studied population. In many studies, ocular involvement in sarcoidosis has been overestimated mainly because study populations have been collected from eye clinics and because the study criteria have included ocular findings or symptoms that do not require treatment or monitoring. In a screening setting, asymptomatic ocular sarcoidosis has been detected in only 2–5%. 0–1% of the screened sarcoidosis patients have required treatment. For these reasons, ocular screening in sarcoidosis seems generally of little value. Patients with sarcoidosis who present with ocular symptoms should be screened for ocular sarcoidosis in a timely manner because they are at high risk of ocular disease.

Although asymptomatic ocular sarcoidosis is rare, screening has been recommended for patients with sarcoidosis to rule out ocular inflammation. In 2020, the American Thoracic Society (ATS) recommended baseline eye exams in sarcoidosis because ocular inflammation may be common in sarcoidosis, screening is not harmful nor burdensome, and treatment can be beneficial in maintaining vision (1). The ATS screening recommendation increased screening referrals to Tays Eye Centre, Tampere University Hospital, Tampere, Finland in 2020–2021, even though the pandemic was ongoing, and the clinical experience was that asymptomatic ocular sarcoidosis is rarely seen (1, 2). Therefore, a registry study and a literature review were conducted to better understand the value of ocular screening in sarcoidosis (2).

The rate of ocular involvement in sarcoidosis varies widely, between 2–80% in different studies (2–7). Length of follow-up affects the cumulative rate of ocular inflammation in sarcoidosis and provides one explanation for differences in study results. One study has evaluated the incidence of ocular inflammation in sarcoidosis in an American veteran population, where the incidence of ocular inflammation over a 3-year period was 8% (5). Most commonly, ocular inflammation is diagnosed during the first year after the diagnosis of systemic sarcoidosis (8, 9). The rate of ocular sarcoidosis also depends on the population. The risk of ocular sarcoidosis is especially high in Japan, where for example uveitis occurs in up to 41% of patients with sarcoidosis (3). African-Americans may also have a high risk of developing ocular sarcoidosis but not all results are consistent regarding their risk (4, 7, 10).

Ocular sarcoidosis has been screened in three studies, in which asymptomatic intraocular inflammation was found in 2–5% of patients (2, 11, 12). In the study by Sunshine et al., asymptomatic intraocular inflammation was detected in 2 of 42 patients with histopathologically confirmed sarcoidosis, and neither required treatment (11). In the screening study from Tays Eye Centre, 262 patient records were reviewed: 172 asymptomatic patients were specifically screened for ocular sarcoidosis and 90 patients without any inflammatory symptoms underwent an untargeted comprehensive ocular exam. Two patients (0.8%) had asymptomatic intraocular inflammation that was high enough to require treatment (2). In a screening study by Lee et al., none of the 27 asymptomatic patients required treatment. In their study, 45% of screened patients had ocular symptoms. Two of the 22 patients with symptoms had active intraocular inflammation necessitating treatment (13). Pooling these numbers together, 2 of 331 asymptomatic patients were treated for ocular sarcoidosis. To find one asymptomatic inflammation that required treatment, as many as 165 patients were screened.

Registry studies may underestimate a prevalence because occult diseases are not revealed without screening asymptomatic patients (5). Screening studies estimate disease prevalence better than registry studies, but they also catch clinically insignificant conditions (14). For example, in the screening study by Lee et al., 12% had signs of intraocular inflammation, and more than half of the patients presented with some finding related to ocular sarcoidosis but only 2 were treated (13).

Studies that investigate data from eye centers may overestimate the rate of ocular involvement in sarcoidosis because only some of the patients with sarcoidosis, particularly patients with symptoms, will visit the eye clinic. An example of an overestimate can be created from our registry data. In the population served by Tampere University Hospital, there should have been approximately 480 new sarcoidosis cases during 2014–2021 (15). During 2014–2021, altogether 568 people who had a recorded ICD-10 code for sarcoidosis were treated at Tays Eye Centre. We found confirmation of the diagnosis of sarcoidosis in the electronic charts for 400 patients. During 2014–2021, 97 patients were treated for ocular sarcoidosis (2). Thus, the rate of ocular sarcoidosis was 24% for patients with chart-confirmed sarcoidosis and visiting the Tays Eye Centre, and lower, when unconfirmed cases or the cumulative incidence of sarcoidosis are considered.

Ocular conditions included in the study criteria significantly impact the rate of reported ocular sarcoidosis (Table 1). Study results may be misleading if they report both harmful and harmless conditions in their overall rates. Inclusion of common but less harmful conditions, such as conjunctival nodules detected in up to 59% of patients with sarcoidosis, or cataract in up to 38%, increase the rate of ocular involvement in sarcoidosis (13, 26). For example, in the study by Morimoto et al., one of the recorded ocular findings was visual disturbance which occurred in 20% of patients with sarcoidosis, which potentially affected the overall rate of ocular involvement (55%) in their data (3). Similarly, if we included visual disturbances in our results at Tays Eye Centre, the rate of eye involvement would be 28% instead of 2% although none of the patients experiencing vision problems were diagnosed with ocular sarcoidosis. If we included dry eyes in our study criteria, the rate of ocular sarcoidosis would be 13% in our screening study although none of the patients with dry eye symptoms required follow-up (2).

**Table 1:** Included ocular conditions, rate of ocular involvement, and rate of uveitis in different studies.

	Types of ocular conditions included	Symptomatic patients included	Rate of ocular involvement	Rate of uveitis
Birnbaum et al. (5)	Uveitis, scleritis, orbital involvement	Yes	8%	N/A
Morimoto et al. (3)	Uveitis, visual disturbance, secondary glaucoma, optic nerve, conjunctiva, lacrimal gland, orbital involvement	Yes	55%	41%
Obenauf et al. (16)	Uveitis, orbital or adnexal involvement, lacrimal gland involvement	Yes	38%	N/A
James et al. (17)	Uveitis, conjunctivitis, conjunctival follicles, scleral plaques, cataract	Yes	28%	20%
Ungprasert et al. (18)	Uveitis, dry eyes, conjunctival nodules, scleritis, conjunctivitis, lacrimal gland or eyelid involvement, optic neuritis	Yes	7%	4%
Jabs and Johns (19)	Uveitis, lacrimal gland or conjunctival involvement, secondary glaucoma, cataract, eyelid nodules, iris nodules, band keratopathy, scleral plaque, optic nerve involvement	Yes	26%	19%
Atmaca et al. (20)	“Anterior and posterior involvement,” conjunctival and eyelid involvement	Yes	13%	N/A
Lee et al. (21)	Uveitis	Yes	21%	21%
Jackson et al. (22)	Uveitis, lacrimal gland enlargement	Yes	15%	12%
Evans et al. (4)	Uveitis, adnexal involvement, dry eyes	Yes	80%	12%
Sheu et al. (23)	Uveitis	Yes	35%	35%
Khanna et al. (24)	Uveitis, conjunctival nodule, lid and lacrimal gland involvement	Yes	29%	N/A
Dróbecka et al. (25)	Eyelid involvement, uveitis, optic disc edema	Yes	18%	N/A
Sunshine (11)	Uveitis	No	5%	5%
Joukainen et al. (2)	Uveitis, iris nodules, conjunctival, orbital or eyelid involvement, optic nerve involvement	No	2%	2%
Lee et al. (13)	Conjunctival nodules, lacrimal gland involvement, uveitis	Yes	N/A	13%

Also, the rate of complications in ocular sarcoidosis varies mainly depending on the included ocular conditions. Only one patient (0.3%) in our screening study had developed a uveitis-related complication by the time of screening visit, without any impact in their vision (2). In long-term follow-up, with long-term high-dose steroid treatment, and including both symptomatic and asymptomatic eyes, ocular complications are observed often, especially in uveitis. Cataracts are seen in 62%, secondary glaucoma in 28%, and macular edema in up to 23% in intraocular inflammation related to sarcoidosis (27–29). Chronic or severe sarcoidosis-related ocular inflammation may cause vision loss (28, 29), but not all sarcoidosis-related ocular findings lead to impaired vision, such as conjunctival nodules (13). During the past decades, visual prognosis has improved in ocular sarcoidosis likely due to improved surgical techniques especially in the field of cataract surgery, better inflammatory management, and with advances in antirheumatic treatment (19, 27–29). In 2022, Suzuki et al. reported that only 6% of eyes with ocular sarcoidosis (22 of 323) had irreversible vision loss, mostly due to secondary glaucoma (27). In 1999, the rate of vision loss was much higher, 46%, and 5% of patients lost vision below 20/120 in both eyes (28). Earlier, in 1986, as many as 26% of patients with ocular sarcoidosis lost vision below 20/120 (19). No study has compared treatment outcomes in patients with and without ocular symptoms at diagnosis.

To sum up, rate of ocular involvement in sarcoidosis varies between 2–80% depending mainly on the included ocular conditions, study setting, and population. Low rates occur in a screening setting and high rates occur when including symptomatic patients, primary ocular sarcoidosis, non-inflammatory ocular conditions, and in high-risk populations (2–7, 17, 18). A good way to make a general estimate of the overall prevalence is to calculate a pooled prevalence if the reported results vary. The pooled prevalence estimated from the studies included in the ATS review is 15%, which is much lower than the average prevalence (26%) calculated by the ATS (1) (Table 2). Thus, there was a methodological error in the ATS review (1). In the current analysis, the pooled prevalence of ocular sarcoidosis is 14% among the larger studies (n > 500) included in the ATS review (3, 5–7, 10, 30). Among the small studies (n < 200), the pooled prevalence is as high as 40% (4, 16, 19, 20, 22, 24, 25), explaining the difference between the reported average rate in the ATS review (26%) and the calculated pooled prevalence (15%) in this review (1). One study published in 1974 was excluded from the current analysis because the original paper was not available (23). It is good to note that some of the included studies in the ATS review overestimated the rate of ocular sarcoidosis, as explained in this article and previously by Lee et al. (13).

**Table 2:** Rate of ocular sarcoidosis in different studies.

	Population	Patients with sarcoidosis	Reported ocular involvement, *n* of patients	Rate of ocular involvement
Birnbaum et al. (5)	USA	15,130	1,256	8%
Judson et al. (10)	USA	1,256	287	23%
Baughman et al. (6)	USA	1,587	465	29%
Morimoto et al. (3)	Japan	996	546	55%
Baughman et al. (7)	USA	736	87	12%
Obenauf et al. (16)	USA	532	202	38%
James et al. (17)	UK	442	123	28%
Ungprasert et al. (18)	USA	345	23	7%
Jabs and Johns (19)	USA	183	47	26%
Atmaca et al. (20)	Turkey	139	18	13%
Lee et al. (21)	Korea	104	22	21%
Jackson et al. (22)	UK	82	12	15%
Evans et al. (4)	USA	81	65	80%
Sheu et al. (23)	Taiwan	55	19	35%
Khanna et al. (24)	India	48	14	29%
Dróbecka et al. (25)	Poland	33	6	18%
Overall		21,749	3,192	
Pooled prevalence				15%

Studies including patients with primary ocular sarcoidosis should not guide recommendations for ocular screening in sarcoidosis. Ocular sarcoidosis is diagnosed before systemic sarcoidosis in up to 80% of patients (8, 18, 28). Systemic sarcoidosis is routinely screened in bilateral granulomatous, anterior uveitis, which may present with iris nodules and anterior synechiae; in intermediate or panuveitis with snowballs or strings of pearls; and in uveitis with periphlebitis, multifocal chorioretinitis, or posterior granuloma(s) (31). Systemic sarcoidosis is considered also in inflammatory orbital conditions, or when granulomas are detected on the conjunctiva or eyelids (9). If sarcoidosis is suspected, the patient is asked about their extraocular symptoms (32). Screening panel for asymptomatic systemic sarcoidosis includes at least chest imaging, serum ACE and/or LZM measurement, serum lymphocyte count, and tuberculin test or interferon-gamma releasing assay to rule out tuberculosis (31).

Majority of patients with ocular sarcoidosis present with symptoms (1, 5, 26), further reducing the value of ocular screening in sarcoidosis. Ocular sarcoidosis may present with a wide variety of symptoms including pain, photophobia, lacrimation, redness, blurriness of vision, floaters, eyelid swelling, or even diplopia (9, 13). Studies including patients with ocular symptoms should not guide the future recommendations for screening. The US screening studies did not find any evidence to support screening of asymptomatic sarcoidosis patients (11, 13). After completing our screening study, we stopped screening asymptomatic sarcoidosis patients at Tampere University Hospital, Finland (2). We should conduct similar screening studies in different patient populations and report in detail which findings were related to inflammation, which findings required follow-up and treatment, and which findings caused ocular symptoms. Further screening studies should be performed in potentially high-risk populations such as Japan (3), Korea (21), and among African-Americans (4, 7).

The benefits of screening asymptomatic ocular sarcoidosis seem very uncertain because majority of the patients present with ocular symptoms (1, 5, 26), patients are commonly first diagnosed with ocular sarcoidosis (8, 18, 28), and because asymptomatic ocular sarcoidosis requiring treatment is rare (2, 11, 13). All patients with sarcoidosis who present with ocular symptoms should be screened for ocular sarcoidosis in a timely manner because they are at high risk of ocular disease.

## Author contributions

SL: Conceptualization, Methodology, Writing – original draft.
